# Restoration of hippocampal growth hormone reverses stress-induced hippocampal impairment

**DOI:** 10.3389/fnbeh.2013.00066

**Published:** 2013-06-14

**Authors:** Caitlin M. Vander Weele, Christopher Saenz, Junmei Yao, Susana S. Correia, Ki A. Goosens

**Affiliations:** Department of Brain and Cognitive Sciences, McGovern Institute for Brain Research, Massachusetts Institute of TechnologyCambridge, MA, USA

**Keywords:** growth hormone, hippocampus, stress, fear, gene therapy, conditioning

## Abstract

Though growth hormone (GH) is synthesized by hippocampal neurons, where its expression is influenced by stress exposure, its function is poorly characterized. Here, we show that a regimen of chronic stress that impairs hippocampal function in rats also leads to a profound decrease in hippocampal GH levels. Restoration of hippocampal GH in the dorsal hippocampus via viral-mediated gene transfer completely reversed stress-related impairment of two hippocampus-dependent behavioral tasks, auditory trace fear conditioning, and contextual fear conditioning, without affecting hippocampal function in unstressed control rats. GH overexpression reversed stress-induced decrements in both fear acquisition and long-term fear memory. These results suggest that loss of hippocampal GH contributes to hippocampal dysfunction following prolonged stress and demonstrate that restoring hippocampal GH levels following stress can promote stress resilience.

## Introduction

Stress is defined by a constellation of responses that occur when the body's ability to cope with physical or psychological demands is exceeded (McEwen and Wingfield, 2007). Stress exposure can vary in duration, and it is clear that stress “load,” defined by both the length of exposure as well as the number of stressors present, plays a role in determining the consequences of stress (Juster et al., 2011). Short-term stress is thought to recruit adaptive responses that promote coping and resilience. However, the mechanisms for driving adaptive change may be difficult to maintain in the face of repeated challenge, and maladaptations can occur when stress is prolonged (McEwen, 1998). For example, high stress load is a risk factor for the development of numerous types of affective mental illness, particularly those involving fear and anxiety (Mazure, 1995; Belanoff et al., 2001; Lederbogen et al., 2011). Despite an abundant literature on the effects of stress in the brain, most studies have focused on the effects of acute stress. Thus, the mechanisms that lead to maladaptations following chronic stress exposure remain unclear.

While there are many brain regions that are altered by stress and mediate stress-associated changes in behavior, the hippocampus is the region in which the effects of stress are best characterized. The hippocampus plays a role in many types of memory (Jeneson and Squire, 2012), and is also linked to affective regulation (Bangasser and Shors, 2007; Goosens, 2011). Acute stress can both enhance and impair hippocampal function. For example, acute stress can increase (Shors et al., 2001) or decrease (Chen et al., 2008) hippocampal dendritic spine density. Acute stress can also enhance (Shors, 2001) or impair (de Quervain et al., 1998) hippocampus-dependent cognition, an effect that may depend on the level of arousal attained during the stress (Diamond et al., 2007). In contrast, chronic stress generally produces dendritic retraction in hippocampus (Watanabe et al., 1992; Magarinos and McEwen, 1995; Vyas et al., 2002; Sandi et al., 2003), and impairs performance on hippocampus-dependent memory tasks (Nishimura et al., 1999; Pawlak et al., 2005; Kleen et al., 2006). These changes are thought to be mediated, in part, by stress hormone-induced downregulation of growth factors, such as brain-derived neurotrophic factor, in neurons (Lakshminarasimhan and Chattarji, 2012).

Growth hormone (GH) is released into the circulating blood stream by the pituitary, but it is also synthesized by the hippocampus and other brain regions (Nyberg and Burman, 1996; Sun et al., 2005) where it may act as a local neuromodulator. Within the hippocampus, application of exogenous GH is sufficient to induce synaptic plasticity (Zearfoss et al., 2008). Exogenous GH also facilitates hippocampal synaptic transmission (Mahmoud and Grover, 2006; Molina et al., 2012) and hippocampus-dependent eyeblink conditioning is associated with enhanced GH protein synthesis in hippocampal cells (Donahue et al., 2002). Interestingly, hippocampal GH levels are stress-sensitive: GH gene transcription is regulated by glucocorticoid stress hormones (Treacy et al., 1991) and GH protein levels are increased one day after an acute stress exposure (Donahue et al., 2006). These findings suggest that higher levels of hippocampal GH may promote hippocampal function, but these studies are correlational. Here, we examine hippocampal GH following chronic stress and explore the relationship between GH and stress-related changes in hippocampal function by using viral-mediated gene transfer to manipulate GH levels in stressed and unstressed rats prior to training on one of two hippocampus-dependent behavioral tasks. While the hippocampus is a complex structure, and it plays a role in many aspects of memory, we focused on two well-characterized yet distinct aspects of hippocampal function: the role of the hippocampus in forming contextual representations (Maren et al., 2013), assayed by contextual fear conditioning, and the role of the hippocampus in maintaining a memory “trace” over a delay interval (Shors, 2004), assayed by auditory trace conditioning.

## Materials and methods

### Subjects

All experiments used adult male Long–Evans rats (225–275 g, Taconic, Germantown, NY), housed individually (20–22.2°C; 12 h light-dark cycle, 0700 lights on). Rodent chow and water was provided *ad libitum*. Stressed and unstressed rats were housed in separate cubicles. All procedures were in accordance with the US National Institutes of Health (NIH) Guide for the Care and Use of Laboratory Animals and were approved by the MIT Institutional Animal Care and Use Committee, and the Animal Care and Use Review Office (ACURO) of the Army Research Office.

### Immobilization stress

Immobilization stress was administered for 4 h per day for 10 (contextual fear conditioning experiment) or 14 (trace fear conditioning experiment) consecutive days. Rats were placed in Decapicone plastic bags (Braintree Scientific; Braintree, MA), which were secured at the tail to keep the bagged rat in an upright position. Stress occurred in an isolated lab room, separate from all behavioral testing space. All stress sessions were performed between 1000 and 1600. Unstressed control rats were handled daily for 30 s.

### Growth hormone ELISA

Hippocampi were homogenized in homogenization buffer (2% HALT protease cocktail and 0.15% NP-40 in PBS; 6 ul buffer per 1 mg of tissue) using a LabGEN 125 homogenizer (Cole-Parmer; Vernon Hills, IL) for 8–10 s on ice. After 5 min of incubation on ice, tubes were spun at 18,000 g for 20 min at 4°C and the supernatant was transferred to a new tube. The resulting solution was assayed as per manufacturer's protocol (Millipore; Billerica, MA).

### Amplicon construction

The rat presomatotropin gene was cloned as an 818 bp HindIII cut fragment from the p-RGH1 plasmid (Seeburg et al., 1977), provided by Dr. Douglas Weigent (University of Alabama at Birmingham), into the HindIII cloning site of the HSV amplicon plasmid pα22GFP (Kaufer et al., 2004), in which a bicistronic HSV-based promoter simultaneously drives expression of a transgene from the α-4 promoter and enhanced green florescent protein (eGFP) from the α-22 promoter. The pα22GFP plasmid was used as a control.

### Virus preparation

Virus was generated using standard methods (Lim and Neve, 2000). Briefly, plasmids were amplified to generate endotoxin-free DNA, which was transfected into 2–2 cells. The next day, cells were superinfected with 5*dl*1.2 helper virus. After two days, the cells were frozen and thawed three times, sonicated to release infectious viral particles, and centrifuged to clear the medium of cell debris. The resulting supernatant was twice passaged onto 2–2 cells. After the final sonication and centrifugation, the supernatant was purified on a sucrose gradient, pelleted, and resuspended in 10% sucrose in D-PBS. Aliquots of each amplicon were stored at −80°C until use. Amplicon titers ranged from1 to 4 × 10^8^ IU/ml. Within each experiment, control and GH-expressing viral titers were similar titers.

### Protein (western) immunoblot

Vero cells were plated in 6 cm dishes using standard methods (Lim and Neve, 2000). Purified HSV virus was used to infect cells at multiplicities of infection ranging from 0 to 0.2. After three days, cells were harvested and homogenized. Protein was loaded on to gels for electrophoretic transfer. Membranes were incubated, in succession, with the following primary antibodies overnight at 4°C: 1:5000 rabbit anti-GH (National Hormone and Peptide Program, NIDDK), 1:500 mouse anti-GFP (Roche, Indianapolis, IN), 1:1000 mouse anti-Actin (Millipore; Billerica, MA). Following incubation in secondary antibody, immunoreactivity was visualized using chemiluminescent detection.

### Stereotactic virus delivery

Surgery was performed 18–24 h following the final handling or immobilization stress session. Rats were anesthetized (with either Nembutal at 65 mg/kg, or a ketamine:xylazine:acepromazine cocktail at 100:100.10 mg/kg, i.p.) and mounted in a stereotaxic frame. Small holes were drilled for intra-cranial placement of the injector within the dorsal hippocampus: A/P −3.3 mm, M/L ±2.0 mm, D/V −3.2 mm, relative to brain surface and bregma (Paxinos and Watson, 2005). Virus was infused with either pulled glass pipettes or 33 g stainless steel bevel needles attached to a 10 μl Hamilton syringe (Hamilton Company, Reno, NV). The pipettes or syringes were mounted in stereotaxic barrel holder, and the rate of virus delivery was controlled by a syringe pump (Harvard Apparatus, Holliston, MA). Virus was infused at 0.1 μl/min for 20 min (2 μl total volume per hemisphere). Injectors remained in the brain for 10 min before being withdrawn. Incisions were closed with wound clips and Ketoprofen (1 mg, s.c.) was administered for pain and inflammation.

### Pavlovian fear conditioning

All behavioral testing commenced 72 h after stereotactic viral delivery, a time point corresponding to maximal transgene expression with HSV-based viral vectors (Lim and Neve, 2000). Fear conditioning experiments were conducted in a modified chamber (MED Associates; St. Albans, VT) housed in a sound-attenuating cubicle. For auditory trace fear conditioning, rats were placed in individual chambers in a novel context (house and room lights on, 1% acetic acid, grid floors) for 5 min before receiving tone (20 s, 2 kHz, 85 dB)-footshock (1 s, 0.85 mA) pairings, with the stimuli separated by a 35 s trace interval. A 3 min inter-trial interval (ITI) was used. Long-term contextual fear memory was assessed 24 h later, when the rats were returned to the chambers for a 5 min context extinction test. Long-term auditory fear memory was measured 24 h later; the rats were placed in the chamber configured as a novel context (room and house lights off, 0.3% Pine Sol odor, white Plexiglas floor and wall inserts). Rats were allowed 3 min to habituate to the chamber before 4 tones (85 dB, 2 kHz) were presented with ITIs of 3 min 35 s. For contextual fear conditioning, rats were placed in a novel context (house and room lights on, 0.3% Pine Sol, grid floors) for 3 min before receiving 3 unsignaled footshocks (2 s, 0.5 mA) separated by a 90 s inter-stimulus interval. Long-term contextual memory was measured 24 h later, when the rats were returned to the context for an 8 min context test. Infrared video was recorded throughout all sessions. Freezing was measured offline using commercial software (VideoFreeze, MedAssociates, St. Albans, VT).

### Histology

Following completion of the experiment, animals were anesthetized with an overdose of isoflurane and the brains were removed from the cranium. Brains from animals that experienced behavioral testing were bisected along the midline. The dorsal hippocampus was dissected from one hemisphere, placed in a sterile eppendorf tube, and flash frozen in dimethylbutane on dry ice. The tissue was stored at −80°C until GH levels were quantified by ELISA to compare expression in stressed and unstressed control animals. The other hemisphere was placed in 4% paraformaldehyde for 72 h then transferred to a 30% sucrose/4% paraformaldehyde solution for a minimum of 3 days. Hemispheres extracted for each treatment were counterbalanced. Fixed tissue was cut into coronal sections on a cryostat (40 μm) and mounted on slides. Sections were assessed for GFP florescence. Animals with incorrect placements were excluded from all analyses. Brains used to assay viral expression of GH levels were placed in a brain matrix and sliced into coronal sections (2 mm thick; 1 mm on either side of the injection site, visible from the dorsal brain surface). The dorsal hippocampus was dissected from each section and flash frozen. The tissue was stored at −80°C until GH levels were quantified by ELISA.

## Results

**Figure 1:**
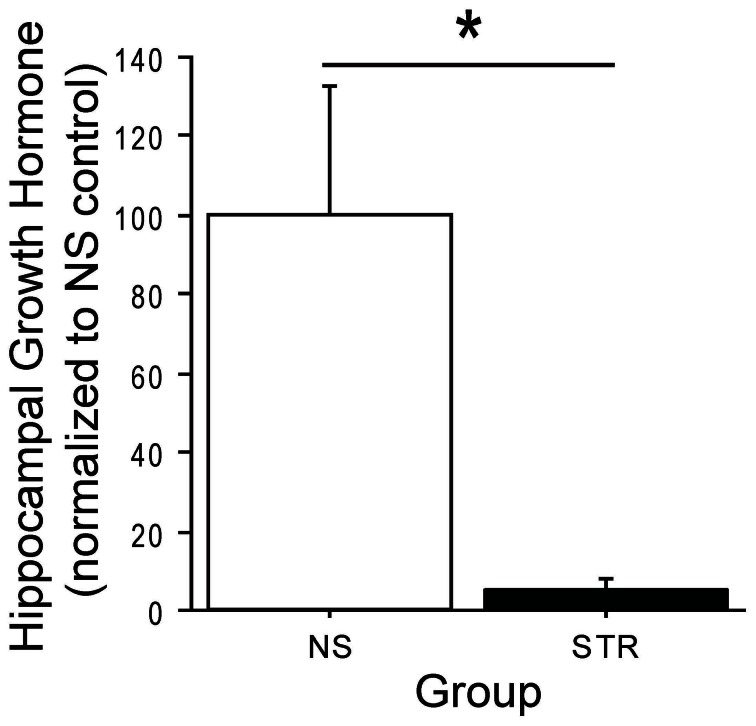
**Chronic stress reduces hippocampal growth hormone (GH).** Hippocampal GH levels were assayed seven days after a two week period of immobilization stress (STR; *n* = 4) or handling (NS; *n* = 4). GH levels were expressed as a percentage, relative to the NS control average. GH was significantly lower in the hippocampi of stressed rats relative to unstressed rats. ^*^Indicates *p* < 0.05.

It has been shown that an acute stress exposure leads to elevated hippocampal GH (Donahue et al., 2006) and enhanced performance on delay eyeblink conditioning (Shors et al., 1992), a hippocampus-dependent task. However, it is not known how GH is affected by chronic stress. To address this, we quantified GH levels in the dorsal hippocampus of rats after either 14 consecutive days of immobilization stress (STR) or daily handling (no stress, or NS). Hippocampal GH was dramatically downregulated following chronic stress (Figure 1; group: *F*_(1, 6)_ = 8.29, *p* < 0.05). This finding reaffirms that acute and chronic stress can produce very different effects on the brain.

**Figure 2:**
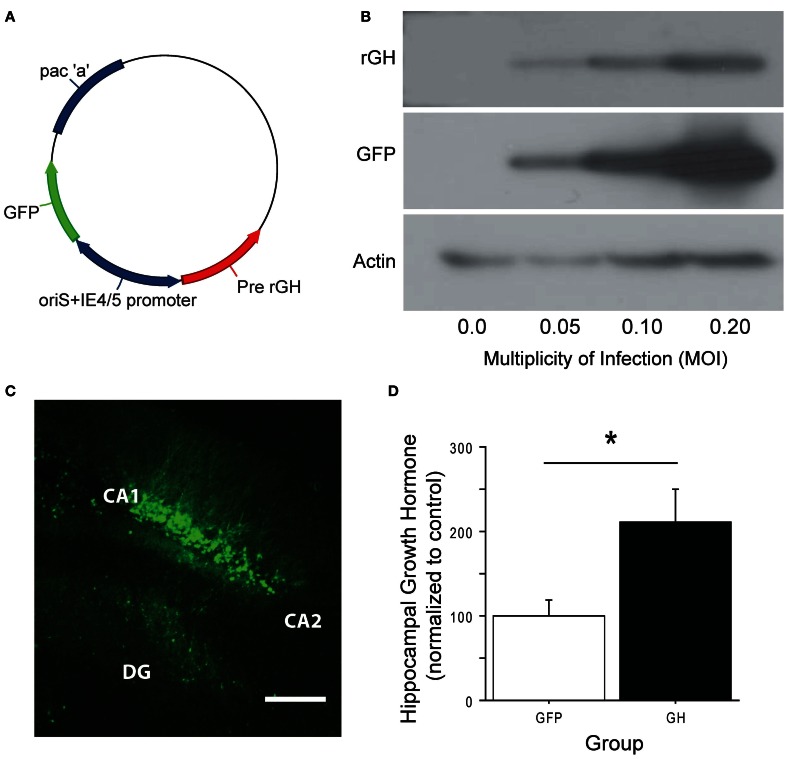
**Construction of an HSV-1 viral vector to overexpress GH. (A)** The full-length gene for presomatotropin was cloned into an HSV-1 amplicon under the control of the HSV α-4 promoter. eGFP was co-expressed via the HSV α-22 promoter. **(B)** GH protein expression was confirmed *in vitro*. Vero cells were infected with GH virus at increasing MOIs. As the MOI increased, progressively higher levels of both GH and eGFP were detected. **(C)** The viral vector was infused into the dorsal hippocampus of rats. A representative infection, showing high levels of expression in pyramidal cells of CA1, and sparse infection in the granule cell layer of the dentate gyrus, is shown. Scale bar = 100 microns. **(D)** GH protein expression was quantified in infected dorsal hippocampal slices four days following virus delivery. GH levels were expressed as a percentage, relative to the NS control average. Viral overexpression of GH led to an approximate doubling of GH protein. ^*^Indicates *p* < 0.05.

To explore whether restoration of hippocampal GH following chronic stress would rescue stress-related impairment on hippocampus-dependent tasks, we constructed an HSV-1 based amplicon in which the full-length gene for rat presomatotropin (rGH), the precursor molecule for GH (Seeburg et al., 1977), was co-expressed with green fluorescent protein (GFP) under the control of bicistronic viral promoters (Figure 2A). This amplicon, as well as a control amplicon expressing only GFP, was packaged into replication-defective HSV viral vectors. To confirm that the viral vector was working as designed prior to *in vivo* application, we used Western blot with an antibody against rGH and showed that the GH viral vector produced GH protein in infected, dissociated cell cultures, and higher levels of infection led to higher levels of expressed protein (Figure 2B). We then used these vectors to infect the dorsal hippocampus of rats (Figure 2C). The majority of infected cells were pyramidal neurons in areas CA1 and CA2 of the dorsal blade of the hippocampus, with varying levels of infection in the granule cell layer of the underlying dentate gyrus. Rats with infection in the overlying cortex were excluded from the experiment. After four days for post-operative recovery, at a time point that corresponds to peak HSV-mediated gene expression, we quantified the expression of GH protein in unstressed animals that received intra-hippocampal infusions of either the GH or control viral vector (Figure 2D). Overexpression of GH led to an approximate doubling of the GH protein in the infected dorsal hippocampus (Figure 2D; group: *F*_(1, 10)_ = 7.84, *p* < 0.05), suggesting that our infection parameters could approximate physiological GH levels when used in stressed rats where GH levels are nearly depleted (Figure 1).

**Figure 3:**
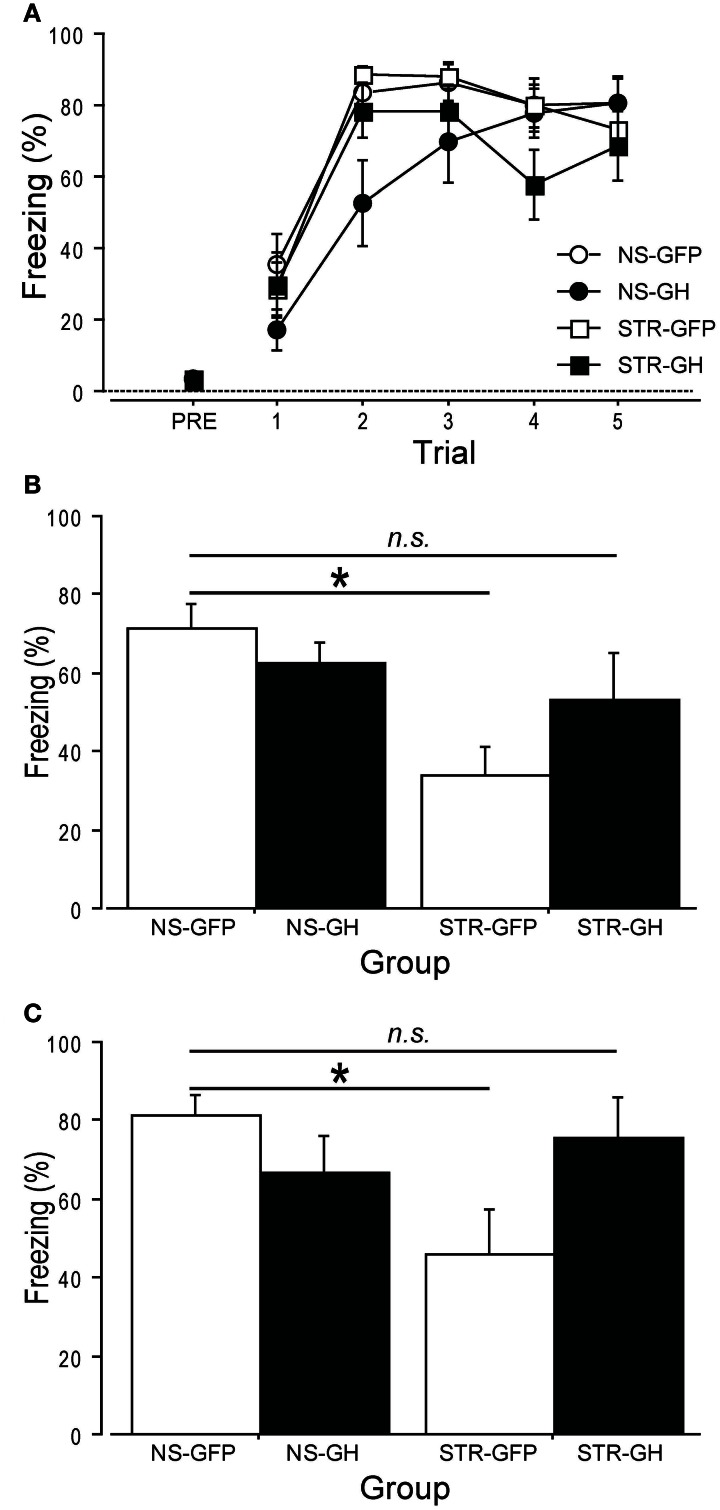
**Overexpression of hippocampal GH rescues stress-related impairments in auditory trace conditioning.** Two weeks of daily immobilization stress (STR) or handling (NS) were administered to rats. Twenty-four hours after the last session, GH or GFP virus was infused bilaterally into the dorsal hippocampus. (NS-GFP, *n* = 9; NS-GH, *n* = 7; STR-GFP, *n* = 7; STR-GH, *n* = 6). **(A)** After three days of recovery, auditory trace conditioning was administered. Neither stress nor GH administration affected conditional freezing during training. **(B)** Long-term contextual fear memory was measured the next day by returning rats to the conditioning context for 5 m. Stress impaired contextual fear memory, and intra-hippocampal GH expression partially reversed this impairment. **(C)** Long-term auditory fear memory was measured the following day by placing the rats in a novel context and presenting four tones in the absence of footshock. Stress impaired auditory fear memory, and intra-hippocampal GH expression fully reversed this effect. ^*^Indicates *p* < 0.05 in a *post-hoc* comparison.

We first examined the role of GH in chronic stress-related changes in auditory trace fear conditioning, a hippocampus-dependent task (Raybuck and Lattal, 2011). Rats were repeatedly exposed to daily immobilization stress (STR) or handling (NS). One day later, rats received intra-hippocampal infusions of either GH or GFP virus. After three days for recovery, rats were subjected to auditory trace fear conditioning. Over the following two days, long-term contextual fear memory and auditory trace fear memory were assessed. Stress did not affect the rapid acquisition of auditory trace fear conditioning (Figure 3A; stress: *F*_(1, 25)_ = 0.02, *p* = ns), and this was not differentially impacted by GH expression (Infusion × Stress interaction: *F*_(1, 25)_ = 0.17, *p* = ns). In contrast, the effects of GH expression on long-term contextual and trace auditory fear memory did depend on stress (Figure 3B; Infusion × Stress interaction: *F*_(1, 25)_ = 3.22, *p* = 0.08; and Figure 3C; Infusion × Stress interaction: *F*_(1, 25)_ = 5.99, *p* < 0.05). Whereas rats in the STR-GFP group showed lower levels of conditional freezing than rats in the NS-GFP group, rats in the STR-GH group displayed levels of conditional freezing that were statistically indistinguishable from those displayed by rats in the NS-GFP group (Figures 3B,C, *post-hoc* comparisons). These results suggest that the impairments in hippocampal function following chronic stress can be attributed to the loss of hippocampal GH.

**Figure 4:**
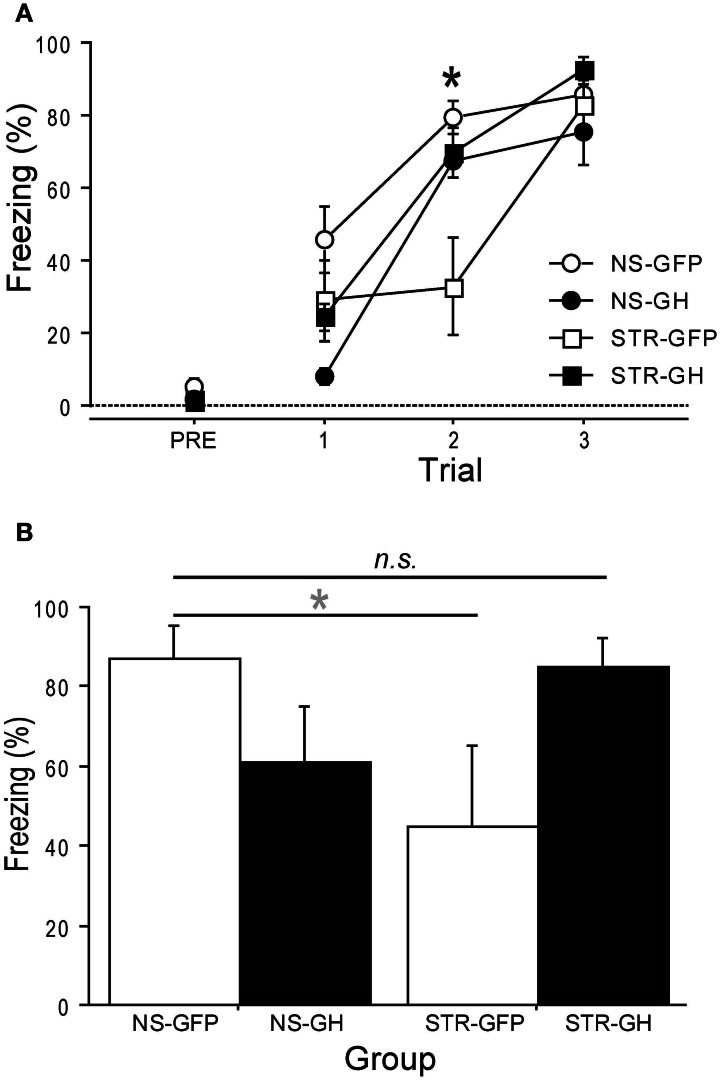
**Overexpression of hippocampal GH rescues stress-related impairments in contextual conditioning.** Ten days of daily immobilization stress (STR) or handling (NS) were administered to rats. Twenty-four hours after the last session, GH or GFP virus was infused bilaterally into the dorsal hippocampus. (NS-GFP, *n* = 4; NS-GH, *n* = 3; STR-GFP, *n* = 4; STR-GH, *n* = 3). **(A)** After three days of recovery, contextual fear conditioning was administered. Stress slowed contextual fear acquisition (^*^Indicates *p* < 0.05 in *post-hoc* comparisons between STR-GFP and other groups), and this was prevented by intra-hippocampal GH. In contrast, intra-hippocampal GH had no effect in unstressed control rats. **(B)** The next day, the rats were returned to the context for an 8 m context extinction session. Stress impaired long-term contextual fear memory, and this impairment was rescued by expression of GH in the dorsal hippocampus. In contrast, intra-hippocampal GH tended to produce a mild impairment of long-term contextual fear memory in unstressed control rats. ^*^Indicates *p* < 0.1 in a *post-hoc* comparison.

To further investigate this, we also examined the role of GH in stress-related changes in foreground contextual fear conditioning, a hippocampus-dependent task (Anagnostaras et al., 2001). Rats were repeatedly exposed to daily immobilization stress (STR) or handling (NS). One day later, rats received intra-hippocampal infusions of either GH or GFP virus. After recovering for three days, rats were subjected to contextual fear conditioning. Long-term contextual memory was measured the next day. The effects of stress on contextual fear acquisition were dependent on the type of virus that had been infused in the hippocampus (Figure 4A; Stress × Infusion interaction, *F*_(1, 10)_ = 1.35, *p* < 0.01): rats in the STR-GFP group displayed slower acquisition than rats in the STR-GH group (*post-hoc* comparisons). In contrast, rats in the NS-GFP and NS-GH groups acquired fear at virtually identical rates (*post-hoc* comparisons). Similar effects of GH were observed for long-term contextual memory (Figure 4B; Stress × Infusion interaction, *F*_(1, 10)_ = 5.29, *p* < 0.05): intra-hippocampal GH rescued the memory-impairing effects of stress, leading to conditional freezing levels indistinguishable from NS-GFP controls (*post-hoc* comparisons). However, intra-hippocampal GH in unstressed controls produced a mild impairment in conditional freezing, relative to NS-GFP controls (*p* = 0.09; *post-hoc* comparison). These results provide further support for the idea that a loss in hippocampal GH contributes to stress-related impairment in hippocampal function.

## Discussion

Here, we show that chronic stress induces a profound and lasting downregulation of GH in the dorsal hippocampus. Rats that experienced chronic stress also exhibited significant impairment on two hippocampus-dependent tasks. It is tempting to speculate that a stress-induced loss of hippocampal GH may contribute to stress-related impairment in hippocampal function, though we did not explicitly test whether a loss of GH is sufficient to lead to impairment of hippocampal function. When GH levels were increased in stressed animals using viral-mediated gene transfer, the rats did not exhibit any stress-related decrements in performance. This shows that, regardless of the root cause of stress-related impairment of hippocampal function, restoration of GH after stress termination is sufficient to reverse these changes. While both of the tasks that we used to assay hippocampus-dependent behaviors involve fear, it is highly unlikely that the role of hippocampal GH is specific to tasks involving fear. Indeed, high levels of hippocampal GH are associated with better performance on tasks requiring other facets of hippocampal function, such as working memory (Ramis et al., 2013). Thus, GH likely plays a broad role in hippocampal function.

It is interesting to speculate about the mechanisms engaged by GH signaling following viral expression in the stressed brain. Chronic GH has been shown to upregulate the NR2B subunit of the NMDA receptor (Le Greves et al., 2002), which could lead to enhanced hippocampal function by prolonging neuronal excitation and enhancing long-term plasticity (Tang et al., 1999). While there are no studies to explicitly demonstrate that GH promotes dendritic spine formation, given the tight correlations between stress-related changes in GH levels and stress-related changes dendritic spines (acute stress enhances both, and chronic stress decreases both, in hippocampus), there may also be an unrecognized relationship between the two. Increases in spine density or NR2B expression in hippocampus could promote neuroplastic changes at hippocampal synapses, and learning-related plasticity in hippocampus is thought to underlie context conditioning (Marschner et al., 2008; Kheirbek et al., 2013) and trace conditioning (Thompson et al., 1996; Moyer et al., 2000). GH may also boost hippocampal function by restoring normal levels of neurogenesis (Ransome and Turnley, 2008) following stress (Vollmayr et al., 2003).

Because GH can potentiate hippocampal synaptic plasticity, one might hypothesize that overexpression of GH would lead to enhancement of hippocampal function. Interestingly, overexpression of GH in the hippocampus of unstressed animals had minimal effect on contextual or trace fear conditioning. For animals subjected to contextual fear conditioning, there was a mild trend for unstressed rats to have impaired long-term contextual fear memory when GH was overexpressed in hippocampus. This may be due to an occlusion effect, whereby GH transiently saturates plasticity in the hippocampus such that synapses may not be further potentiated by learning. However, overexpression of GH clearly did not produce a broad occlusion of further hippocampus-dependent learning. An alternative hypothesis to explain the lack of occlusion is that GH may regulate its own expression, and viral expression of recombinant GH could have downregulated expression of endogenous GH, though that does not appear to be the case (Figure 2D). Regardless, these results support GH as a novel target for pharmacological intervention following stress, and suggest that interventions that boost GH signaling in hippocampus after stress could promote stress resilience (Fleshner et al., 2011).

## Author contributions

Caitlin M. Vander Weele collected and prepared samples for the GH ELISAs, performed surgeries for the trace conditioning experiment and the ELISA experiments, and collected and analyzed data for the contextual fear conditioning experiment. Christopher Saenz performed surgeries and collected data for the trace fear conditioning experiment. Junmei Yao collected and analyzed data for the GH ELISA. Susana S. Correia planned and executed the Western blot experiment to test viral expression of GH. Ki A. Goosens designed experiments and generated viral constructs, acquired and analyzed data for the trace conditioning experiment, and wrote the manuscript.

### Conflict of interest statement

The authors declare that the research was conducted in the absence of any commercial or financial relationships that could be construed as a potential conflict of interest.
